# Synthesis of Ordered Mesoporous Zr-Al Composite Oxides with Excellent Structural and Textural Properties and Extremely High Stability

**DOI:** 10.3390/ma13133036

**Published:** 2020-07-07

**Authors:** Feng Yu, Shinan Bi, Tonghui Liu, Dahai Pan, Shuwei Chen, Xiaoliang Yan, Binbin Fan, Ruifeng Li

**Affiliations:** Research Centre of Energy Chemical & Catalytic Technology, College of Chemistry and Chemical Engineering, Taiyuan University of Technology, Taiyuan 030024, China; yufeng@tyut.edu.cn (F.Y.); bishinan0451@link.tyut.edu.cn (S.B.); liutonghui0023@163.com (T.L.); csw603@163.com (S.C.); yanxiaoliang@tyut.edu.cn (X.Y.); fanbinbin@tyut.edu.cn (B.F.)

**Keywords:** mesoporous materials, Zr-Al composite oxide, thermal stability, hydrothermal stability, cooperative co-assembly

## Abstract

Ordered mesoporous Zr-Al composite oxide materials (denoted as OMZA-x) with different Zr contents have been synthesized by a solvent evaporation-inducing self-assembly procedure associated with a thermal treatment at 100 °C. A cooperative co-assembly process of amphiphilic triblock copolymer F127 molecules and inorganic hydroxyl species originated from the hydrolysis of Zr and Al precursors was proposed to explain the synthesis of OMZA-x. Compared to ordered mesoporous alumina prepared without introducing Zr species, the resultant OMZA-x exhibited a much more ordered mesostructure combined with a distinct increase in the pore volume and specific surface area. The highly homogenous doping of Zr into the mesopore walls together with the formation of Zr-O-Al bonds can effectively enhance the thermal and hydrothermal stability of OMZA-x. For instance, the ordered mesostructure and excellent textural properties of OMZA-6 prepared with the optimum atomic ratio of Al to Zr of 6 could be well maintained even after a high-temperature treatment at 1000 °C for 1 h or a hydrothermal treatment at 100 °C for 6 h.

## 1. Introduction

Since the first invention of M41s series of silica materials [1,2], ordered mesoporous materials possessing large pore volume and high specific surface area as well as tunable mesoporous structures have attracted extensive attention for the development of high-efficiency catalysts using in the conversion of macromolecules [3], and numerous non-silicon-based ordered mesoporous materials with excellent structural, textural, and surface properties have been successively synthesized [4,5,6,7]. Compared to ordered mesoporous silica materials, ordered mesoporous alumina materials as catalysts or catalyst supports exhibit much more broad application prospects in various petrochemical processes, including hydrodesulfurization, cracking, and hydrocracking of petroleum feedstocks [8,9,10,11,12], because of the thoughtful combination of both the chemical properties of alumina and ordered mesoporous characteristics. At present, ordered mesoporous alumina materials are generally synthesized via two main strategies. One is the nano-casting method using pre-formed ordered mesoporous silica or carbon as hard templates [13,14]. The other is the soft-template self-assembly process in which the surfactant supramolecular aggregates are used as the ordered mesostructure-directing templates [15,16,17]. Compared to the first strategy involving more than one impregnation step combined with high preparation cost, the soft-template self-assembly procedure is more convenient. Unfortunately, the typical soft-template synthesis recipes for ordered mesoporous silica materials could not be effectively enabled to prepare the alumina analogues, because of the complicated hydrolysis-condensation behavior of aluminum precursors [18,19,20].

The evaporation induced self-assembly (EISA) method is another soft-template self-assembly approach for the successful preparation of ordered mesoporous materials, especially ordered mesoporous metal oxides [7,8,21,22,23]. Via such a method, a series of ordered mesoporous alumina materials with different mesostructures and tunable mesoporous sizes have also been successfully prepared by adjusting the hydrophilic-hydrophobic properties of surfactants or introducing suitable catalysts into the initial synthesis solution [8,24,25,26]. During the EISA process, the utilization of volatile non-aqueous solvents (such as anhydrous ethanol and tetrahydrofuran) can effectively slow down the hydrolysis-condensation rate of aluminum precursors, and a self-assembly ordered organic-inorganic composite micelle structure can be induced by gradually increasing the surfactant concentration during the solvent evaporation process. After calcination to decompose the surfactant micelles existing in the mesochannels of alumina, the alumina materials with ordered mesostructure can be finally obtained. Clearly, the use of EISA procedure can synthesize ordered mesoporous alumina materials in a facile manner, since the hydrolysis-condensation conditions of aluminum precursors do not need to be strictly controlled. However, the high-temperature thermal stability of ordered mesoporous alumina materials prepared by the EISA procedure is still not satisfactory. When the thermal treatment temperature reaches above 800 °C, the aluminum species existing within the mesopore walls will begin to aggregate, sinter, and transform into the crystalline alumina, resulting in the collapse of ordered mesoporous structure along with a distinct reduction in the pore volume and specific surface area [15,27]. Therefore, the poor high-temperature thermal stability severely restricts the practical applications of ordered mesoporous alumina under high temperature reaction conditions. Moreover, for applications that require ordered mesoporous alumina to be exposed in a hydrothermal environment for a long time, the mesostructural stability is very important, which is still another great challenge severely hindering the practical applications of ordered mesoporous alumina.

The recent investigations have confirmed that the doping of suitable heteroatoms (such as Si, Mg, and Zr) into the matrix of alumina can effectively remove the hydroxyl groups, anions and/or cation holes existing on the surface of alumina, consequently, the thermal stability of alumina materials can be significantly improved [28,29,30,31,32]. Zirconia, as a transition metal oxide, features acid-base properties and oxidation-reduction quality; thus, the thoughtful combination of both zirconia and ordered mesoporous alumina allows the fabrication of advanced support materials for the development of high-efficiency catalysts [33]. The synthesis of Zr-doped alumina with ordered mesostructure has been reported in the previous literature, and the obtained materials display much more structural stability than that of pure ordered mesoporous alumina [29]. However, there is no detailed report on the effects of the doping amount of Zr on the mesostructure and textural properties as well as stability of obtained materials. In this paper, ordered mesoporous Zr-Al composite oxide materials (denoted as OMZA-x) with different atomic ratios of Al to Zr were synthesized via a solvent EISA method associated with a thermal treatment at 100 °C. The resultant OMZA-x exhibit a well-defined ordered 2D hexagonal mesostructure, a large pore volume, a high specific surface area, a uniform mesoporous size, and a highly homogenous distribution of Zr and Al at the atomic level. More importantly, the homogenous incorporation of suitable Zr atoms into the mesopore walls and the formation of Zr-O-Al bonds can remarkably enhance the thermal and hydrothermal stability of obtained materials. The effects of the doping amount of zirconium on the synthesis of OMZA-x materials and their performance boost were investigated, and the possible synthesis mechanism was provided.

## 2. Experimental Section

### 2.1. Chemicals

Triblock copolymer F127 (EO_106_PO_70_EO_106_) was obtained from Sigma-Aldrich, St. Louis, MO, USA. Zirconium oxychloride, aluminum isopropoxide, citric acid, 37 wt % hydrochloric acid, and anhydrous ethanol were purchased from Tianjin Chemical Reagent Co., Tianjin, China. All of these analytically graded chemicals were directly used.

### 2.2. Material Synthesis

**Figure 1:**
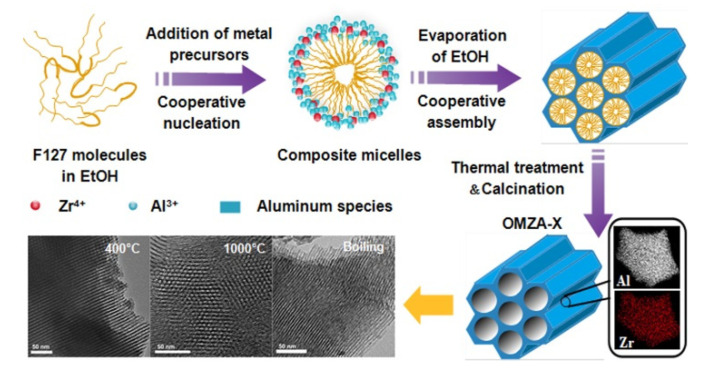
Schematic illustration for the synthesis of ordered mesoporous Zr-Al composite oxide materials (OMZA-x).

The ordered mesoporous Zr-Al composite oxides were synthesized by a similar method reported in our previous works [28,34], as shown in Figure 1. In a typical synthesis, 1.6 g of 37 wt % concentrated hydrochloric acid, 0.4 g of citric acid, and 3.2 g of F127 were successively dissolved in 20 mL anhydrous ethanol, followed by the slow addition of aluminum isopropoxide (0.016 mol) and a required amount of zirconium oxychloride (0.0016, 0.002, 0.0027, 0.004, and 0.008 mol, respectively) under stirring. After being strongly stirred at 32 °C for 24 h, the resulting clear sol was transferred into a dish to successively undergo solvent evaporation treatment at 45 °C for 48 h and thermal treatment at 100 °C for 24 h. The obtained solid products were calcined at 400 °C for 5 h to remove the organic template, and were named as OMZA-x, where x denotes the atomic ratio of Al to Zr in the initial synthesis solution.

As a reference, ordered mesoporous alumina (OMA) sample was also prepared using the same procedure as that of OMZA-x excluding the introduction of Zr precursor during the synthesis process, and was named as OMA.

### 2.3. Characterization

Power X-ray diffraction (XRD) analysis was taken on a Shimadzu XRD-6000 diffractometer (Shimadzu Corporation, Kyoto, Japan) using Ni-filtered Cu Kα (0.154 nm) radiation. Transmission electron microscopy (TEM) images were obtained on a JEOL JEM-2100F microscope (Japan Electronics Corporation, Tokyo, Japan) with an operation voltage of 200 kV. Inductively coupled plasma atomic emission spectrometry (ICP-AES) was performed on a Thermo iCAP 6300 spectrometer (Spectro, Kleve, Germany). Before measurement, the sample was dissolved using a mixed solution of nitric acid and hydrofluoric acid. Elemental mapping analysis was conducted on a Hitachi Model S-4800 high-resolution fluorescence-emission scanning electron microscopy instrument (Hitachi Limited, Tokyo, Japan) with an acceleration voltage of 25 kV. N_2_ sorption analysis was conducted on a Quantachrome analyzer (Quantachrome Instruments, Boynton Beach, FL, USA) at −196 °C. Before measurement, the sample was degassed under vacuum at 180 °C for 8 h. The specific surface area was calculated with the use of Brunauer-Emmett-Teller (BET) method, the pore size distribution was derived from the adsorption branches of the isotherm using Barrett-Joyner-Halenda (BJH) method, and the total pore volume (V_p_) was determined according to the adsorbed N_2_ amount at a relative pressure of 0.99. Fourier transformed infrared (FT-IR) spectra were obtained on a Shimadzu IR Affinity-1 spectrometer (Shimadzu, Kyoto, Japan). For each spectrum, 32 scans were collected in the range from 400 to 4000 cm^−1^ at a resolution of 4 cm^−1^. ^27^Al magic angle spinning nuclear magnetic resonance (^27^Al MAS NMR) measurements were performed on a Bruker AVANCE III 600 MHz spectrometer (Bruker, Karlsruhe, Germany) operating at a resonance frequency of 156.47 MHz with a recycle delay of 1s. The aluminum nitrate solution (1.0 mol/L) was used as external reference.

### 2.4. Stability Evaluation

The high-temperature thermal stability and hydrothermal stability of samples OMZA-x and OMA were evaluated by a thermal treatment at 1000 °C in air for 1 h and a boiling water treatment in a closed autoclave at 100 °C for different time, respectively.

## 3. Results and Discussion

**Figure 2:**
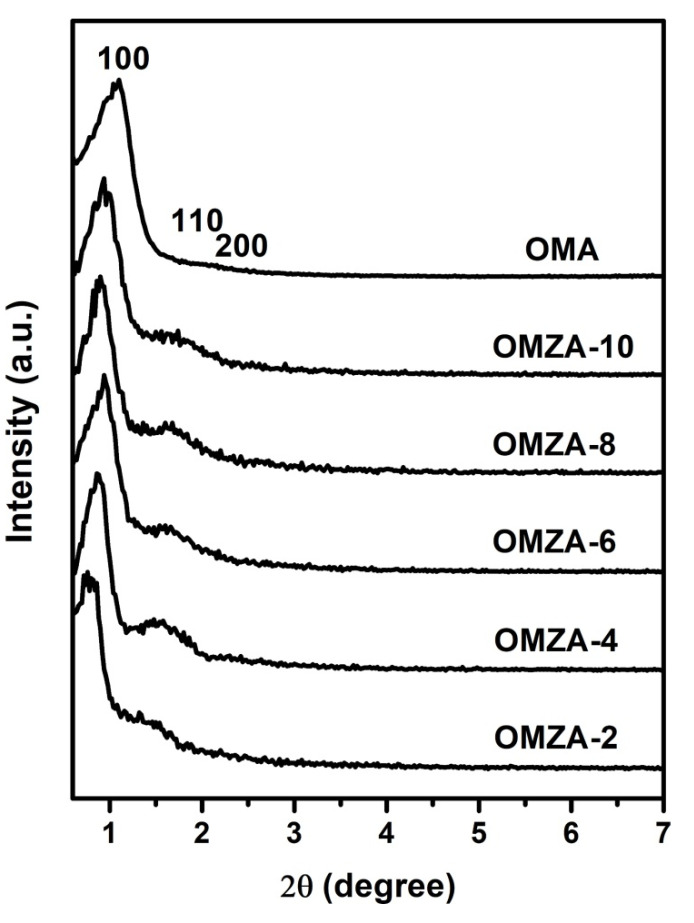
Small-angle X-ray diffraction (XRD) patterns of samples OMZA-x and OMA.

**Figure 3:**
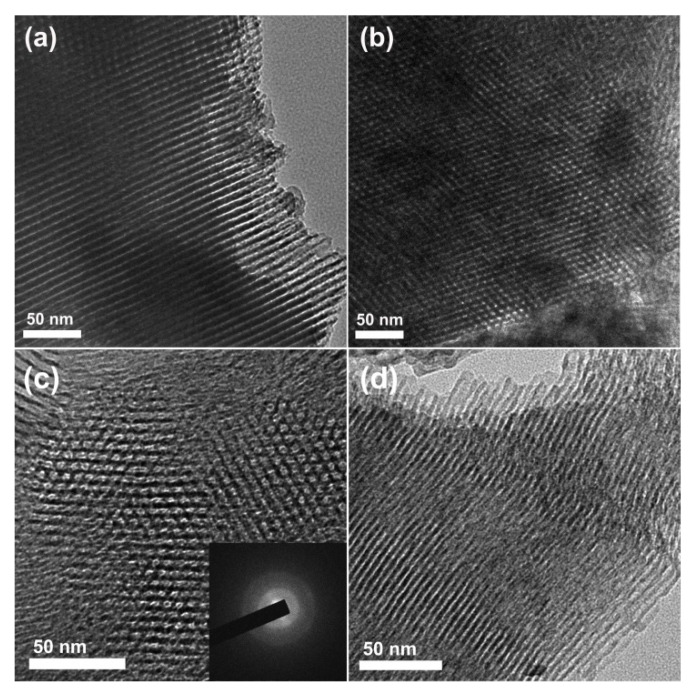
Transmission electron microscopy (TEM) images of sample OMZA-6 viewed along the (110) (**a**) and (100) (**b**) directions, TEM image (**c**) of OMZA-6 thermally treated at 1000 °C for 1 h, and TEM image (**d**) of OMZA-6 hydrothermally treated at 100 °C for 6 h, respectively.

For samples OMZA-x and OMA, the evidence for the presence of ordered mesostructure is presented by the small-angle XRD patterns. As can be seen in Figure 2, all samples exhibited two well-resolved diffraction peaks in a 2θ range from 0.5° to 3°, characterizing an ordered mesostructure in the pore arrangement [8,15,35]. Taking the sample OMZA-6 for example, a very strong diffraction peak at 0.94° and one weak broad peak around 1.64° are clearly obvious. According to the TEM observations (Figure 3), such two diffraction peaks can be indexed to the (100) reflection and the overlapping of the (110) and (200) reflections, respectively, indicating that the sample OMZA-6 has an ordered two-dimensional (2D) hexagonal mesostructure. Interestingly, compared to sample OMA, the samples OMZA-x (except for sample OMZA-2) displayed a narrower full width at half-maximum (FWHM) as judged from the (100) reflection peak and stronger overlapping intensity of the (110) and (200) reflections, corresponding to an obviously increased mesoscopic order. According to the angular positions of (100) reflections, the interplanar spacing (*d*_100_) values of 11.04, 10.27, 9.39, 9.81, 9.39, and 8.03 nm can be calculated for samples OMZA-2, OMZA-4, OMZA-6, OMZA-8, OMZA-10, and OMA, respectively (Table 1). It is worthwhile to note that with the increase in the introducing amount of Zr, the FWHM of the (100) diffraction peaks of samples OMZA-x firstly decreased and then gradually increased, and an opposite variation trend was observed in the overlapping intensity of the (110) and (200) reflections. Among all OMZA-x, the sample OMZA-6 demonstrates the narrowest FWHM of the (100) diffraction peaks and the strongest overlapping intensity of the (110) and (200) reflections, representing the most ordered 2D hexagonal mesoporous structure. The observation results from the small-angle XRD patterns indicate that the suitably introduced Zr species will take part in and promote the cooperative self-assembly of surfactant F127 molecules and Al species to effectively construct a much more ordered mesostructure. However, the excessive introduction of Zr species into the synthesis solution will hinder the efficient self-assembly between inorganic Al species and organic template. Consequently, the mesoscopic structure order of samples OMZA-x (x < 6) gradually decreased with increasing the introducing amount of Zr.

**Figure 4:**
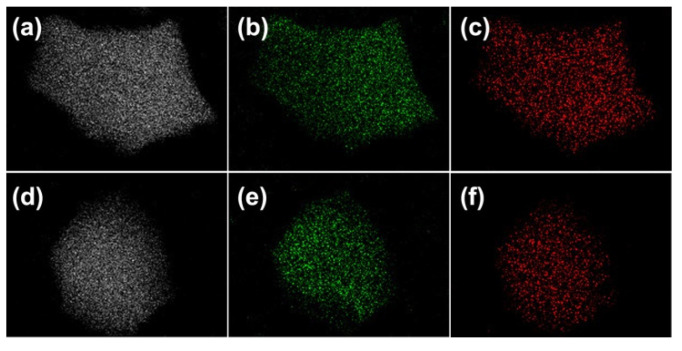
Elemental analysis mapping showing the distribution of Al (**a**,**d**), O (**b**,**e**), and Zr (**c**,**f**) for two different regions of the sample OMZA-6.

The TEM images of sample OMZA-6 further provide an observation of ordered mesostructure. As shown in Figure 3a,b, the ordered parallel cylindrical shaped mesopores along (110) orientation and hexagonally packed honeycomb-like mesopores along the (100) orientation were clearly obvious, signifying the presence of typical 2D hexagonal mesoporous structure with uniform mesoporous size. The distance between two neighboring mesoporous channels is 10.5 nm, which is well consistent with the calculated unit cell parameter (*a*) by the small-angle XRD analysis combined the equation of *a* = 2*d*_100_/3^1/2^. Interestingly, under the high resolution TEM mode, a strong signal of Zr was clearly observed in the energy-dispersive X-ray (EDX) spectrum of sample OMZA-6 (not shown), verifying that a large amount of Zr atoms exist within the mesopore walls. At three different positions randomly selected, the atomic ratios of Al to Zr measured from EDX analysis were 5.8, 6.1, and 6.3, respectively, all which are very close to the atomic ratio (6.1) measured by ICP-AES analysis and the theoretical atomic ratio (6) for the synthesis of sample OMZA-6. This indicates that almost all Zr species added into the synthesis solution have been finally incorporated into the mesopore walls of sample OMZA-6; more importantly, Zr and Al species existing within the mesopore walls of OMZA-6 can be highly homogenously distributed at an atomic level. Such expected distribution throughout the whole sample was further verified by the elemental mapping images from the scanning electron microscopy with energy dispersive X-ray spectroscopy (SEM-EDX) analysis. As shown in Figure 4, when two different regions are scanned, highly uniform distributions of Al, O, and Zr were found. The calculated atomic ratios of Al to Zr in such two regions randomly selected are 6.0 and 6.2, respectively.

**Figure 5:**
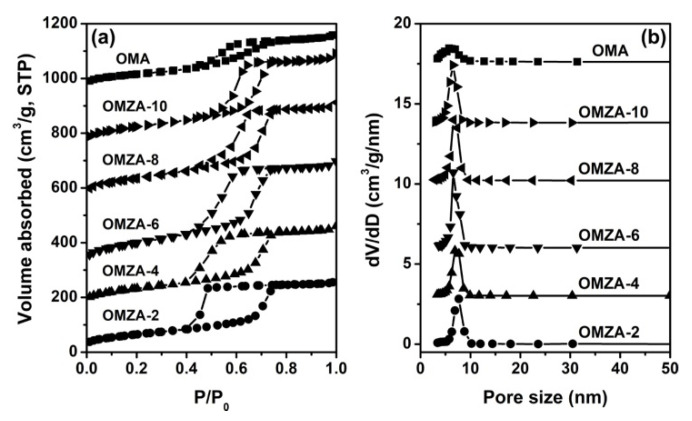
N_2_ sorption isotherms (**a**) and pore size distribution curves (**b**) of samples OMZA-x and OMA. For clarity, in (**a**), the isotherms of OMZA-4, OMZA-6, OMZA-8, OMZA-10 and OMA are offset along the Y axis by 150, 300, 560, 750, and 950 cm^3^/g, respectively. In (**b**), the Y axis values are increased by 3.0, 6.0, 10.2, 13.8, and 17.8 cm^3^/g, respectively.

**Figure 6:**
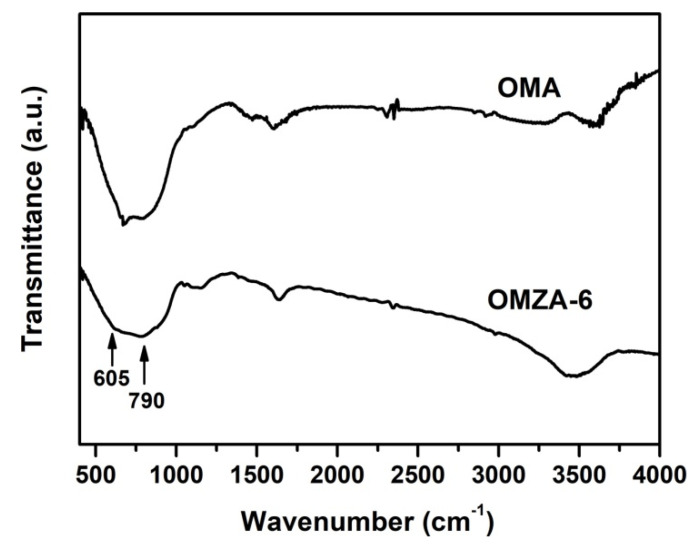
FT-IR spectra of samples OMZA-6 and OMA.

The N_2_ sorption isotherms and the corresponding pore size distribution curves of samples OMA and OMZA-x are shown in Figure 5. The detailed textural properties of these samples are listed in Table 1. From Figure 5a, it can be seen that all samples exhibited typical IV-type isotherms with relatively steep capillary condensation steps, signifying the presence of uniform mesopores [8,26]. It is noteworthy that samples OMZA-x (except for OMZA-2) demonstrated a steeper capillary condensation step than that of sample OMA, corresponding to a much narrower mesoporous size distribution (Figure 5b), which is well in accordance with the characterization result from the small-angle XRD analysis (Figure 2). In addition, compared with sample OMA, with the increase in the introducing amount of Zr, a distinct shift toward higher relative pressures ranging from 0.5 to 0.7 was observed in the isotherms of samples OMZA-x (Figure 5a), indicating samples OMZA-x have a larger average mesoporous diameter than that of sample OMA (Figure 5b and Table 1), and the mesoporous size gradually increases with increasing the introducing amount of Zr. The increase in the mesoporous size could be used to manifest that a large amount of Zr^4+^ ions with larger atomic radius than that of Al^3+^ have been homogenously incorporated into the mesopore walls to form the Zr-O-Al bonds, since the bond length of Zr-O is longer than that of Al with O. The similar observation has also been reported in the previous literatures [28,34]. The formation of Zr-O-Al bonds can be well verified by the FT-IR analysis. Figure 6 presents the FT-IR spectra of representative samples OMZA-6 and OMA. It can be seen that different from sample OMA, a shoulder peak at 605 cm^−1^, associated to the deformation vibration of the Zr-O-Al bond [36,37], can be clearly observed in the low-energy region of the spectrum of sample OMZA-6. Besides the increased mesoporous size, the pore volume and specific surface area of samples OMZA-x are obviously higher than those of sample OMA (Table 1). Considering that the introduced amount of aluminum precursor is same for each sample, the distinct improvement in the textural properties for samples OMZA-x once again verifies that the Zr species originated from the hydrolysis of Zr precursor can take part in and effectively facilitate the cooperative self-assembly of Al species and template F127 molecules. Among all OMZA-x, the OMZA-6 displays the most excellent textural properties. Its pore volume and specific surface area are 0.62 cm^3^/g and 358 m^2^/g, respectively (Table 1). However, further increasing the introducing amount of Zr, a continuous reduction in the pore volume and specific surface area were observed for samples OMZA-4 and OMZA-2. For these two samples, the pore volumes and specific surface areas are 0.45 cm^3^/g and 264 m^2^/g, and 0.40 cm^3^/g and 231 m^2^/g, respectively (Table 1). Evidently, the variation trend in the textural properties for samples OMZA-x is well coincided with their mesoscopic structure order, which can be attributed to the effect of the introducing Zr amount on the synthesis of samples OMZA-x. Interestingly, the introducing amount of Zr can also affect the mesoporous shape of samples OMZA-x, which is reflected in the type of hysteresis loop. For samples OMZA-10 and OMZA-8, their isotherms presented a IV-type curve with a type H1 hysteresis loop, which is the typical characteristic of ordered mesoporous materials with uniform cylindrical shaped channels; while, with an increase in the introducing amount of Zr, a type H2 hysteresis loop was observed in the isotherms of samples OMZA-6, OMZA-4, and OMZA-2, suggesting the presence of cage-neck shaped mesopores. Especially sample OMZA-2, prepared with introducing the highest amount of Zr, showed a very steep desorption step at a relative pressure (P/P_0_) of 0.43, corresponding to a H2(a)-type hysteresis loop. This indicates sample OMZA-2 possesses narrow necks interconnecting the cages. In the case of samples OMZA-4 and OMZA-6, a H2(b)-type hysteresis loop was observed in their isotherms, suggesting that such two samples have similar cage-neck shaped mesopores with broader necks. The transformation of cylindrical mesopore into cage-neck mesopore can be attributed to the influence of introducing Zr amount on the synthesis of samples OMZA-x (Figure 2).

According to the characterization results from XRD, TEM, SEM-Mapping, N_2_ physisorption, and FT-IR analyses, it is clearly obvious that the introduction of an appropriate amount of Zr precursor (zirconium oxychloride) into the synthesis solution exerts a significant influence on promoting the formation ordered mesoporous structure. Here, a triconstituent cooperative co-assembly process was proposed for explaining the synthesis of OMZA-x, as shown in Figure 1. In an anhydrous ethanol solvent with a weak polarity, the triblock copolymer surfactant F127 molecules usually lose their hydrophilic-hydrophobic properties [18,26,38]. Consequently, when inorganic precursors of Zr and Al have not been added into the reaction solution, it is difficult to directly form the micelle phase of surfactant F127 molecules, although the introduction of citric acid and hydrochloric acid containing a certain amount of H_2_O can facilitate the folding of surfactant molecules. After the addition of Zr and Al precursors, the presence of 37 wt % hydrochloric acid as a catalyst plays a significant role in promoting the hydrolysis of these two inorganic precursors to form Zr-OH and Al-OH species, respectively; meanwhile, the added citric acid as a ligand can bond with these hydroxyl species in a bridging or bidentate fashion, resulting in the condensation rate of the formed hydroxyl species being effectively slowed down [15,28]. Under such controlled hydrolysis-condensation conditions, the hydrogen bonding interaction between the hydroxyl groups of Zr and Al and the hydrophilic block (PEO) of surfactant F127 molecules can facilitate the cooperative nucleation and in turn promote the formation of sphere-like organic-inorganic composite micelles, in which the hydrophilic block (PEO) interacting with Zr-OH and Al-OH remains in the corona surrounding the core composed with the hydrophobic block (PPO) of surfactant molecules. During the following solvent evaporation process at a temperature of 45 °C, the progressively increased concentration of organic-inorganic composite micelles impels these composite micelles to self-organize into cylindrical mesostructures and in turn into a thermodynamically stable ordered 2D hexagonal mesostructure. For the subsequent thermal treatment process at 100 °C, such ordered composite micelle mesostructure (i.e., ordered arrangements of cylindrical micelles embedded within the matrices of zirconia and alumina) will undergo further framework shrinkage and wall condensation. During this period, the Zr-OH and Al-OH groups existing within the mesopore walls will experience dehydroxylation and cross-linking together, leading to the formation of Zr-O-Al and Al-O-Al bonds. At last, after calcination to remove the surfactant molecules, the ordered mesoporous Zr-Al composite oxide materials are obtained. Obviously, in the whole self-assembly process, the cooperative nucleation of organic and inorganic species and the formation of composite micelles are the key for the formation ordered mesostructure, which can be realized by enhancing the hydrogen boning interaction between hydroxyl species of Zr and Al and surfactant molecules. For the synthesis solution, the suitable introduction of Zr-OH species from the hydrolysis of Zr precursor can effectively disperse Al-OH species and prevent their polymerization into Al-OH nano-clusters with low hydroxyl contents. As a result, the hydrogen bonding interaction between inorganic hydroxyl species and organic template F127 molecules can be effectively enhanced by retaining a large amount of oligomer Al-OH species with high hydroxyl contents. However, further increasing the introducing amount of Zr precursor, the formed excessive Zr-OH species existing in the reaction solution will obstruct the cooperative self-assembly of Al-OH species and surfactant molecules, leading to a decreased mesoscopic structure order of resultant Zr-Al composite oxides.

**Figure 7:**
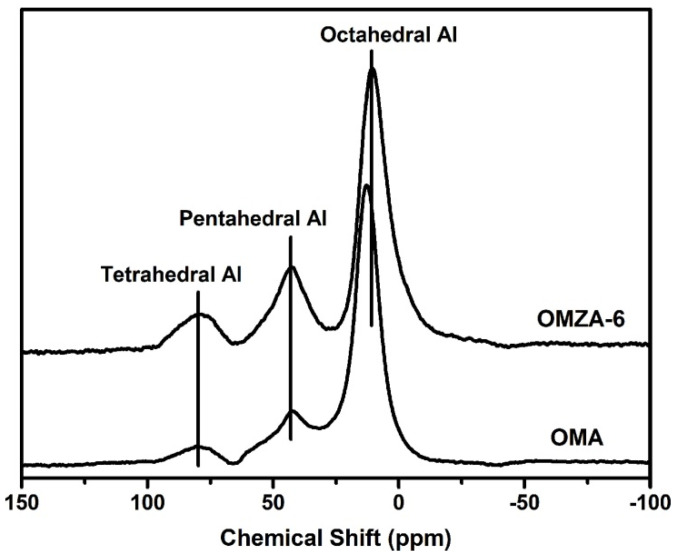
^27^Al MAS NMR spectra of samples OMZA-6 and OMA.

To further underpin the proposed synthesis mechanism, ^27^Al MAS NMR analysis was employed to present the direct evidence for the coordination state of Al species existing within the mesopore walls of OMZA-x, and OMA was a comparison. Figure 7 displays the ^27^Al MAS NMR spectra of representative samples OMZA-6 and OMA. As shown in Figure 7, samples OMZA-6 and OMA display three resonance signals at 10, 41, and 79 ppm, suggesting that three different oxygen environments surround Al sites over these two samples. The signals at 10 and 79 ppm can be assigned to the octa-coordinated non-framework Al species (AlO_6_) and the tetra-coordinated framework Al species (AlO_4_), respectively [26,28]. The appearance of signal at 41 ppm can be attributed to the formation of penta-coordinated Al species (AlO_5_) originating from the distortion of framework aluminum species [15,39,40]. It is worthwhile to note that compared to sample OMA, OMZA-6 exhibited remarkably increased signal intensities for the AlO_4_ and AlO_5_ at the expense of a deduction in the signal intensity for AlO_6_. After classical decomposition and the direct integration for the respective peaks, the calculated proportion of AlO_4_, AlO_5_, and AlO_6_ are 14.7% and 10.0%, 26.5% and 21.0%, and 58.8% and 69%, for samples OMZA-6 and OMA, respectively. Compared to sample OMA, the obviously increase in the contents of framework aluminum species (AlO_4_ and AlO_5_) over sample OMZA-6 indicates that in the self-assembly synthesis solution, the introduction of Zr-OH species can contribute to the highly homogenous distribution of oligomer Al-OH species and effectively prevent the formation of Al-OH nano-clusters mainly composed with octa-coordinated aluminum species due to the polymerization of Al-OH species [26,41]. Consequently, more oligomer Al-OH species with high hydroxyl contents can effectively interact with the hydrophilic block of template F127 molecules by the enhanced hydrogen bonding to form the mesoporous framework of alumina, which can be well responsible for the increased ordered mesostructure of OMZA-6.

**Figure 8:**
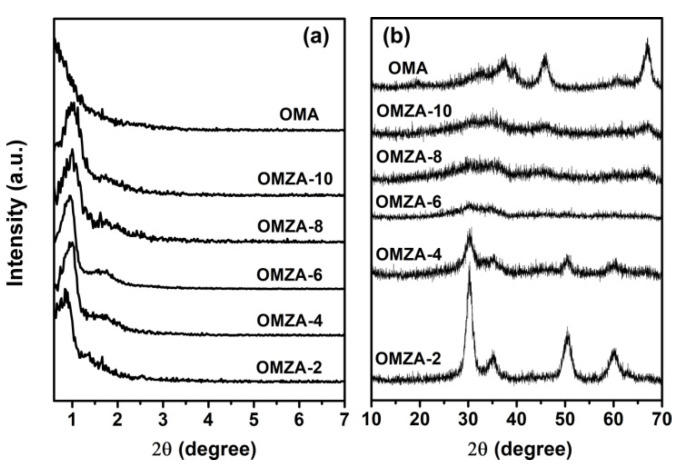
Small- (**a**) and wide- (**b**) angle XRD patterns of samples OMZA-x and OMA after thermal treatment at 1000 °C for 1 h.

**Figure 9:**
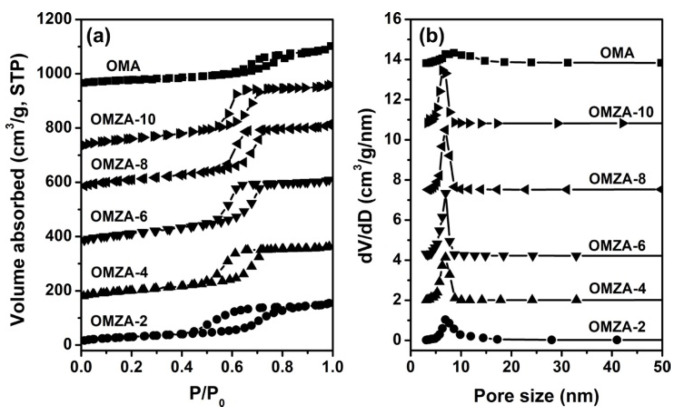
N_2_ sorption isotherms (**a**) and pore size distribution curves (**b**) of samples OMZA-x and OMA after thermal treatment at 1000 °C for 1 h. For clarity, in (**a**), the isotherms of OMZA-4, OMZA-6, OMZA-8, OMZA-10 and OMA are offset along the Y axis by 160, 370, 560, 710 and 950 cm^3^/g, respectively. In (**b**), the Y axis values are increased by 2, 4.2, 7.5, 10.8 and 13.8 cm^3^/g, respectively.

It is well known that the suitable incorporation of second element (such as Si and Mg) into alumina matrix can efficiently restrain the formation of crystal alumina, which plays a crucial role in enhancing the high-temperature thermal stability of ordered mesoporous alumina [29,31,42]. Here, a thermal treatment at 1000 °C for 1 h was selected to investigate the influence of incorporating Zr into the mesopore walls of samples OMZA-x on their thermal stability. Figure 8 presents the small- and wide-XRD patterns of OMZA-x and OMA after thermal treatment. As shown in Figure 8a, no diffraction peak can be discerned in the small-angle XRD pattern of sample OMA, signifying that the ordered mesostructure of sample OMA has been completely destroyed after thermal treatment at 1000 °C for 1 h. Such mesostructural collapse could be attributed to the crystallization of aluminum species occurred within the amorphous mesopore walls of OMA, as confirmed by the wide-angle XRD pattern of sample OMA presented in the Figure 8b. Four well-defined diffraction peaks at around 37.6, 39.5, 45.8, and 66.7°, indexing to (311), (222), (400), and (440) reflections of *γ*-Al_2_O_3_ (JCPDS10-0425), respectively, can be clearly observed. As a result, the thermally treated OMA exhibited an unobvious hysteresis loop and a very broad pore size distribution (Figure 8). By calculation, the pore volume and specific surface area of thermally treated OMA are 0.24 cm^3^/g and 102 m^2^/g (Table 2), respectively. In contrast, after the same high temperature treatment, samples OMZA-x still displayed the well-resolved (100) diffraction peaks in their small-angle XRD patterns, indicating that the mesostructrue of OMZA-x are well preserved. The wide-angle XRD measurement provided the changes of the crystalline structure for thermally treated samples OMZA-x. As shown in Figure 8b, no diffraction peak corresponding to the crystal phase of *γ*-Al_2_O_3_ can be observed for samples OMZA-x, suggesting that the highly homogeneous distribution of Zr (Figure 4) together with the formation of Zr-O-Al bonds (Figure 5 and Figure 6) can effectively prevent the atomic diffusion and sinter of Al species existing within the mesopore walls of OMZA-x, and further suppress their crystallization. However, for samples OMZA-4 and OMZA-2, four characteristic diffraction peaks corresponding to tetragonal zirconia appearing at 2θ = 30, 35, 50 and 60° are discerned in their wide-angle XRD patterns, and the intensity of these four diffraction peaks increases with increasing the zirconium content. The formation of tetragonal zirconia from the sintering and crystallization of the excessive zirconium species existing within the mesopore walls of OMZA-4 and OMZA-2 can be responsible for the partial destruction of ordered mesostructure of these two samples (Figure 8a). Interestingly, no diffraction peak according to the crystal phase of Al_2_O_3_ and ZrO_2_ was discerned in the wide-angle XRD pattern of thermally treated OMZA-6 (Figure 8b), suggesting that the mesopore walls of OMZA-6 is still amorphous phase verified by the selective area electron diffraction (SAED) pattern under TEM model (the inset in Figure 3c). As a result, the sample OMZA-6 still maintains an ordered 2D hexagonal mesostructure even after a high-temperature treatment at 1000 °C for 1 h (Figure 8a). For thermally treated OMZA-6, the ordered arranged hexagonal mesopores and aligned cylindrical mesopores were also clearly obvious in its TEM image (Figure 3c). It is noticeable that the high temperature thermal treatment could result in a rearrangement of the cage-like mesopores towards cylindrical mesopores, which can be verified by the type of hysteresis loop observed in the isotherms of thermally treated samples OMZA-x. As shown in Figure 9, after high-temperature treatment at 1000 °C for 1 h, samples OMZA-x (except for sample OMZA-2 exhibiting a IV-type isotherm with a H2(b)-type hysteresis loop) demonstrated a typical IV-type isotherm with a type H1 hysteresis loop along with a steep capillary condensation step (Figure 9a), corresponding to narrow mesoporous size distributions (Figure 9b). By calculating, the thermally treated OMZA-6 shows a pore volume of 0.42 cm^3^/g and a specific surface area of 228 m^2^/g, which are larger than those of other samples OMZA-x thermally treated under the same conditions (Table 2).

**Figure 10:**
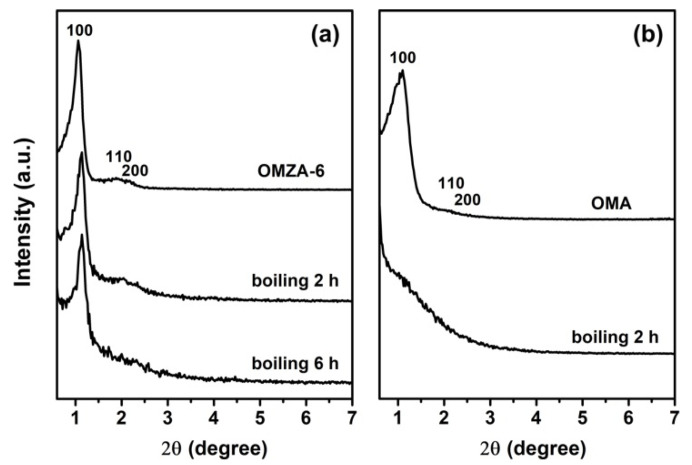
Small-angle XRD patterns of OMZA-6 (**a**) and OMA (**b**) before and after hydrothermal treatment at 100 °C for different time.

**Figure 11:**
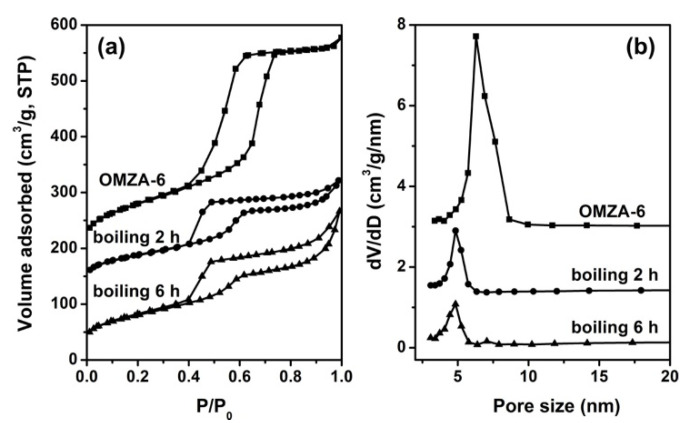
N_2_ sorption isotherms (**a**) and pore size distribution curves (**b**) of sample OMZA-6 before and after hydrothermal treatment at 100 °C for different time. For clarity, in (**a**), the isotherms of untreated OMZA-6 and OMZA-6 hydrothermally treated for 2 h are offset along the Y axis by 200 and 100 cm^3^/g, respectively. In (**b**), the Y axis values are increased by 3.1 and 1.5 cm^3^/g, respectively.

Compared to the high-temperature thermal stability, the hydrothermal stability is much more important, which is still a great challenge severely restraining the practical applications of ordered mesoporous alumina materials. Figure 10 presents the small-angle XRD patterns of representative samples OMZA-6 and OMA before and after boiling water treatment for different time. As shown in the part a of Figure 10, when the hydrothermal treatment is carried out at 100 °C, even the hydrothermal treatment time is prolonged to 6 h, sample OMZA-6 still showed two well-defined diffraction peaks in its small-angle XRD pattern, indicating the ordered mesostructure is maintained to a large extent. Such conclusion can be further confirmed from the observation of TEM image (Figure 3d). In contrast, for sample OMA hydrothermally treated at 100 °C for 2 h, no obvious (100) diffraction peak could be discerned (Figure 10b). This indicates that compared to sample OMZA-6, sample OMA has a less hydrothermal stability, and the long-lasting exposure of OMA to boiling water environment will cause the complete collapse of its mesostructure. The characterization results from N_2_ physisorption analysis (Figure 11) also confirm that sample OMZA-6 has an extremely high hydrothermal stability. It can be seen from Figure 11a that different from untreated sample OMZA-6 only exhibiting a very steep capillary condensation step, after boiling water treatment for 2 h and 6 h, an especial two-step capillary condensation was noticed in the adsorption branches of isotherms. The first step at lower relative pressure ranging from 0.4 to 0.6 can be assigned to the intrinsic ordered mesopores of OMZA-6, which value (4.8 nm) is smaller than that of untreated OMZA-6 due to the shrinkage of mesoporous framework during the hydrothermal treatment process; while the second step at higher relative pressure ranging from 0.85 to 1.0 could be attributed to the formation of interconnected mesopores. During long hydrothermal treatment process times, the partial dissolution of Zr and Al species initially existing within the mesopore walls of OMZA-6 could lead to the generation of disordered mesoporous channel-channel communication combined with an obvious increase in the pore volume and specific surface area (Table 3). Calculations show that, after hydrothermal treatment for 2 h and 6 h, the pore volumes and specific surface areas of OMZA-6 are 0.33 cm^3^/g and 271 m^2^/g, and 0.41 cm^3^/g and 282 m^2^/g, respectively. ICP-AES measurement results further confirmed that compared to Al species, Zr species existing within the mesopore walls of OMZA-6 are more susceptible to dissolution and loss during the hydrothermal treatment process, since the measured atomic ratios of Al to Zr over OMZA-6 hydrothermally treated for 2 h and 6 h were 8.45 and 10.23, respectively.

## 4. Conclusions

Ordered mesoporous Zr-Al composite oxides (OMZA-x) with extremely high hydrothermal stability and thermal stability have been prepared by a triconstituent cooperative co-assembly synthesis pathway. Using such pathway, almost all Zr hydroxyl species originated from the hydrolysis of Zr precursor introduced into the initial synthesis solution can be finally homogenously incorporated into the mesopore walls of resultant OMZA-x. The highly homogenous distribution of Zr and Al at the atomic level and the formation of Zr-O-Al bonds can be responsible for OMZA-x possessing an obviously increased mesostructural order, significantly increased pore volume and specific surface area, and remarkably enhanced thermal and hydrothermal stability. Among all samples OMZA-x, OMZA-6 synthesized with the optimum atomic ratio of Al to Zr of 6 exhibited the most ordered 2D hexagonal mesoporous structure combined with the largest pore volume and highest specific surface area. More importantly, the resultant OMZA-6 displayed superior high-temperature thermal stability and hydrothermal stability. Its ordered mesostructure and excellent textural properties can be well remained even after a thermal treatment at 1000 °C for 1 h or a hydrothermal treatment at 100 °C for 6 h. Such Zr-Al composite oxide nanomaterial may find important applications as catalyst support for the petroleum refinement and the catalytic conversion of heavier petroleum fractions.

## Figures and Tables

**Table 1:** The textural properties of samples OMZA-x and OMA. ^a^

Samples	*d*_100_	*S*_BET_ (m^2^/g)	*V*p (cm^3^/g)	*D*p (nm)
OMZA-2	11.04	231	0.40	7.7
OMZA-4	10.27	264	0.45	6.9
OMZA-6	9.39	358	0.62	6.6
OMZA-8	9.81	312	0.56	6.6
OMZA-10	9.39	303	0.55	6.5
OMA	8.03	257	0.35	6.3

^a^*d*_100_, the *d*_100_ interplanar spacing; *S*_BET_, the specific surface area; *V*_p_, the total volume; *D*_p_, the average mesoporous size.

**Table 2:** The textural properties of OMZA-x and OMA thermally treated at 1000 °C for 1 h. ^a^

Samples	*d*_100_	*S*_BET_ (m^2^/g)	*V*p (cm^3^/g)	*D*p (nm)
OMZA-2	10.50	110	0.24	6.9
OMZA-4	9.39	187	0.33	6.9
OMZA-6	9.39	228	0.42	6.9
OMZA-8	8.83	215	0.41	6.9
OMZA-10	9.01	219	0.40	6.3
OMA	- ^b^	102	0.24	- ^b^

^a^*d*_100_, the *d*_100_ interplanar spacing; *S*_BET_, the specific surface area; *V*_p_, the total volume; *D*_p_, the average mesoporous size. ^b^ The *d*_100_ spacing and average mesoporous size of OMA thermally treated are not calculated due to the complete collapse of ordered mesostructure.

**Table 3:** The textural properties of OMZA-6 before and after hydrothermal treatment at 100 °C for different time. ^a^

Boiling Time (h)	*d*_100_	*S*_BET_ (m^2^/g)	*V*p (cm^3^/g)	*D*p (nm)
0	9.39	358	0.62	6.6
2	7.74	271	0.33	4.8
6	7.74	282	0.41	4.8

^a^*d*_100_, the *d*_100_ interplanar spacing; *S*_BET_, the specific surface area; *V*_p_, the total volume; *D*_p_, the average mesoporous size.

## References

[1] Beck J.S., Vartuli J.C., Roth W.J., Leonowicz M.E., Kresge C.T., Schmitt K.D., Chu C.T., Olson D.H., Sheppard E.W., Mccullen S.B. (1992). A new family of mesoporous molecular sieves prepared with liquid crystal templates. J. Am. Chem. Soc..

[2] Kresge C.T., Leonowicz M.E., Roth W.J., Vartuli J.C., Beck J.S. (1992). Ordered mesoporous molecular sieves synthesized by a liquid-crystal template mechanism. Nature.

[3] Corma A. (1997). From Microporous to Mesoporous Molecular Sieve Materials and Their Use in Catalysis. Chem. Rev..

[4] And M.K., Jaroniec M., And R.R., Joo S.H. (2000). Characterization of Ordered Mesoporous Carbons Synthesized Using MCM-48 Silicas as Templates. J. Phys. Chem. B.

[5] Grosso D., Boissiere C., Smarsly B.M., Brezesinski T., Pinna N., Albouy P.A., Amenitsch H., Antonietti M., Sanchez C. (2004). Periodically ordered nanoscale islands and mesoporous films composed of nanocrystalline multimetallic oxides. Nat. Mater..

[6] Chen P., Yang C., He Z., Guo K. (2019). One-pot facile route to fabricate the precursor of sulfonated graphene/N-doped mesoporous carbons composites for supercapacitors. J. Mater. Sci..

[7] Fu Z., Zhang G., Tang Z., Zhang H. (2020). Preparation and Application of Ordered Mesoporous Metal Oxide Catalytic Materials. Catal. Surv. Asia.

[8] Xu X., Megarajan S.K., Zhang Y., Jiang H. (2020). Ordered Mesoporous Alumina and Their Composites Based on Evaporation Induced Self-Assembly for Adsorption and Catalysis. Chem. Mater..

[9] Yenumala S.R., Kumar P., Maity S.K., Shee D. (2019). Hydrodeoxygenation of karanja oil using ordered mesoporous nickel-alumina composite catalysts. Catal. Today.

[10] Zhang J.Z., Xing X.F., Zhang J.R., Chu J.M., Li Z.X., Zhang Q.Y., Wang R.M., Wu S.H. (2019). Ordered mesoporous alumina with ultra-large pore size catalyze cinnamaldehyde to cinnamyl alcohol with high selectivity. J. Nanopart. Res..

[11] Yuan Q., Duan H., Li L., Li Z., Duan W., Zhang L., Song W., Yan C. (2010). Homogeneously Dispersed Ceria Nanocatalyst Stabilized with Ordered Mesoporous Alumina. Adv. Mater..

[12] Bara C., Plais L., Larmier K., Devers E., Digne M., Lamichumblot A., Pirngruber G., Carrier X. (2015). Aqueous-Phase Preparation of Model HDS Catalysts on Planar Alumina Substrates: Support Effect on Mo Adsorption and Sulfidation. J. Am. Chem. Soc..

[13] Liu Q., Wang A., Wang X., Zhang T. (2006). Ordered crystalline alumina molecular sieves synthesized via a nanocasting route. Chem. Mater..

[14] Wu Z., Li Q., Feng D., Webley P.A., Zhao D. (2010). Ordered Mesoporous Crystalline γ-Al_2_O_3_ with Variable Architecture and Porosity from a Single Hard Template. J. Am. Chem. Soc..

[15] Yuan Q., Yin A.X., Luo C., Sun L.D., Zhang Y.W., Duan W.T., Liu H.C., Yan C.H. (2008). Facile synthesis for ordered mesoporous gamma-aluminas with high thermal stability. J. Am. Chem. Soc..

[16] Huang F., Zheng Y., Cai G., Zheng Y., Xiao Y., Wei K. (2010). A new synthetic procedure for ordered mesoporous γ-alumina with a large surface area. Scripta Mater..

[17] Wu Q., Zhang F., Yang J., Li Q., Tu B., Zhao D. (2011). Synthesis of ordered mesoporous alumina with large pore sizes and hierarchical structure. Micropor. Mesopor. Mater..

[18] Li W., Yue Q., Deng Y., Zhao D. (2013). Ordered mesoporous materials based on interfacial assembly and engineering. Adv. Mater..

[19] Kim Y., Kim C., Choi I., Rengaraj S., Yi J. (2004). Arsenic Removal Using Mesoporous Alumina Prepared via a Templating Method. Environ. Sci. Technol..

[20] Gu D., Schuth F. (2014). Synthesis of non-siliceous mesoporous oxides. Chem. Soc. Rev..

[21] Pal N. (2020). Nanoporous metal oxide composite materials: A journey from the past, present to future. Adv. Colloid Interface Sci..

[22] Cui Y., Lian X.B., Xu L.L., Chen M.D., Yang B., Wu C.E., Li W.J., Huang B.B., Hu X. (2019). Designing and Fabricating Ordered Mesoporous Metal Oxides for CO_2_ Catalytic Conversion: A Review and Prospect. Materials.

[23] Wei J., Ren Y., Luo W., Sun Z.K., Cheng X.W., Li Y.H., Deng Y.H., Elzatahry A.A., Al-Dahyan D., Zhao D.Y. (2017). Ordered Mesoporous Alumina with Ultra-Large Pores as an Efficient Absorbent for Selective Bioenrichment. Chem. Mater..

[24] Grant S.M., Jaroniec M. (2012). Effect of cosolvent organic molecules on the adsorption and structural properties of soft-templated ordered mesoporous alumina. J. Colloid Interf. Sci.

[25] Huang B., Bartholomew C.H., Woodfield B.F. (2014). Facile synthesis of mesoporous γ-alumina with tunable pore size: The effects of water to aluminum molar ratio in hydrolysis of aluminum alkoxides. Micropor. Mesopor. Mater..

[26] Pan D., Chen W., Huang X., Zhang J., Yang Y., Yu F., Chen S., Fan B., Shi X., Cui X. (2018). Solvothermal-assisted evaporation-induced self-assembly of ordered mesoporous alumina with improved performance. J. Colloid Interf. Sci..

[27] Pan D., Guo M., He M., Chen S., Wang X., Yu F., Li R. (2014). Facile synthesis of highly ordered mesoporous chromium–alumina catalysts with improved catalytic activity and stability. J. Mater. Res..

[28] Pan D., Dong Z., He M., Chen W., Chen S., Yu F., Fan B., Cui X., Li R. (2017). Structural and surface properties of highly ordered mesoporous magnesium-aluminium composite oxides derived from facile synthesis. Mater. Chem. Phys..

[29] Wang X., Pan D., Guo M., He M., Niu P., Li R. (2013). Facile synthesis of highly ordered mesoporous alumina with high thermal and hydrothermal stability using zirconia as promoter. Mater. Lett..

[30] Shi J., Zhou Y., Zhang Y., Zhou S., Zhang Z., Kong J., Guo M. (2014). Synthesis of magnesium-modified mesoporous Al_2_O_3_ with enhanced catalytic performance for propane dehydrogenation. J. Mater. Sci..

[31] Zhang Y., Huang B., Mardkhe M.K., Woodfield B.F. (2019). Thermal and hydrothermal stability of pure and silica-doped mesoporous aluminas. Micropor. Mesopor. Mater..

[32] Yuan X., Wang R., Kong L., Guo S. (2019). Fabricating mesoporous silica-modified alumina with high thermal stability. Chem. Phys. Lett..

[33] Lin J., Ma C., Wang Q., Xu Y., Ma G., Wang J., Wang H., Dong C., Zhang C., Ding M. (2019). Enhanced low-temperature performance of CO_2_ methanation over mesoporous Ni/Al_2_O_3_-ZrO_2_ catalysts. Appl. Catal. B Environ..

[34] Pan D., Xu Q., Dong Z., Chen S., Yu F., Yan X., Fan B., Li R. (2015). Facile synthesis of highly ordered mesoporous cobalt–alumina catalysts and their application in liquid phase selective oxidation of styrene. RSC Adv..

[35] Rashidi F., Kharat A.N., Rashidi A., Lima E., Lara V.H., Valente J.S. (2010). Fractal geometry approach to describe mesostructured boehmite and gamma-alumina nanorods. Eur. J. Inorg. Chem..

[36] Moranpineda M., Castillo S., Lopez T., Gomez R., Novaro O. (1999). Synthesis, characterization and catalytic activity in the reduction of NO by CO on alumina-zirconia sol-gel derived mixed oxides. Appl. Catal. B-Environ..

[37] Jabbarnezhad P., Haghighi M., Taghavinezhad P. (2014). Sonochemical synthesis of NiMo/Al_2_O_3_–ZrO_2_ nanocatalyst: Effect of sonication and zirconia loading on catalytic properties and performance in hydrodesulfurization reaction. Fuel Process. Technol..

[38] Bagshaw S.A. (2001). The effect of dilute electrolytes on the formation of non-ionically templated [Si]-MSU-X mesoporous silica molecular sieves. J. Mater. Chem..

[39] Dumeignil F., Rigole M., Guelton M., Grimblot J. (2005). Characterization of Boria−Alumina Mixed Oxides Prepared by a Sol−Gel Method. 1. NMR Characterization of the Xerogels. Chem. Mater..

[40] Xiu T., Wang J., Liu Q. (2011). Ordered bimodal mesoporous boria–alumina composite: One-step synthesis, structural characterization, active catalysis for methanol dehydration. Micropor. Mesopor. Mater..

[41] Haouas M., Taulelle F., Martineau C. (2016). Recent advances in application of ^27^Al NMR spectroscopy to materials science. Prog. Nucl. Mag. Res. Sp..

[42] Del Angel G., Guzmán C., Bonilla A., Torres G., Padilla J.M. (2005). Lanthanum effect on the textural and structural properties of γ-Al_2_O_3_ obtained from Boehmite. Mater. Lett..

